# Age-Related Changes in the Circadian System Unmasked by Constant Conditions^1^,^2^,^3^

**DOI:** 10.1523/ENEURO.0064-15.2015

**Published:** 2015-09-22

**Authors:** Takahiro J. Nakamura, Wataru Nakamura, Isao T. Tokuda, Takahiro Ishikawa, Takashi Kudo, Christopher S. Colwell, Gene D. Block

**Affiliations:** 1 Department of Life Sciences, School of Agriculture, Meiji University, Kanagawa, Kawasaki 214-8571, Japan; 2 Faculty of Pharmaceutical Sciences, Teikyo Heisei University, Tokyo 164-8530, Japan; 3 Department of Psychiatry and Biobehavioral Sciences, University of California, Los Angeles, Los Angeles, California 90024-1759; 4 Laboratory of Oral Chronobiology, Graduate School of Dentistry, Osaka University, Suita, Osaka 565-0871, Japan; 5 Department of Mechanical Engineering, Ritsumeikan University, Shiga, Kusatsu 525-8577, Japan

**Keywords:** aging, imaging, PER2::luciferase, suprachiasmatic nucleus

## Abstract

Circadian timing systems, like most physiological processes, cannot escape the effects of aging. With age, humans experience decreased duration and quality of sleep. Aged mice exhibit decreased amplitude and increased fragmentation of the activity rhythm, and lengthened circadian free-running period in both light-dark (LD) and constant dark (DD) conditions. Several studies have shown that aging impacts neural activity rhythms in the central circadian clock in the suprachiasmatic nucleus (SCN). However, evidence for age-related disruption of circadian oscillations of clock genes in the SCN has been equivocal. We hypothesized that daily exposure to LD cycles masks the full impact of aging on molecular rhythms in the SCN. We performed *ex vivo* bioluminescent imaging of cultured SCN slices of young and aged PER2::luciferase knock-in (PER2::LUC) mice housed under LD or prolonged DD conditions. Under LD conditions, the amplitude of PER2::LUC rhythms differed only slightly between SCN explants from young and aged animals; under DD conditions, the PER2::LUC rhythms of aged animals showed markedly lower amplitudes and longer circadian periods than those of young animals. Recordings of PER2::LUC rhythms in individual SCN cells using an electron multiplying charge-coupled device camera revealed that aged SCN cells showed longer circadian periods and that the rhythms of individual cells rapidly became desynchronized. These data suggest that aging degrades the SCN circadian ensemble, but that recurrent LD cycles mask these effects. We propose that these changes reflect a decline in pacemaker robustness that could increase vulnerability to environmental challenges, and partly explain age-related sleep and circadian disturbances.

## Significance Statement

It is becoming increasingly evident that aging impacts the neural activity rhythms in the central circadian clock in the SCN. However, evidence for age-related disruption of circadian oscillations of clock genes in the SCN has been equivocal. Our data indicate that, at the population level, aging lengthens the free-running period and attenuates the amplitude of population-based PER2::luciferase knock-in rhythms in the SCN. At the single-cell level, aging lengthens the period and reduces the synchrony of single-cell oscillations without reducing their amplitude. These findings were observed in mice housed in prolonged conditions of constant darkness. These data suggest that light-dark cycles mask the effects of aging on SCN cellular clocks and that aging may increase the vulnerability of the SCN circadian ensemble.

## Introduction

In mammals, daily behavioral and physiological rhythms are generated by a complex endogenous timing system involving cell-, tissue-, and organ-level mechanisms (Mohawk et al., 2012). The central circadian pacemaker, the suprachiasmatic nucleus (SCN) in the hypothalamus, is entrained by environmental light-dark (LD) cycles via photosensitive retinal ganglion cells. The SCN synchronizes the rhythms of many other brain regions, along with peripheral tissues, via neural and humoral factors (Welsh et al., 2010; LeGates et al., 2014). Circadian rhythms are generated within mammalian cells by interlocking transcriptional–translational feedback loops involving a family of clock genes (Partch et al., 2014). Briefly, transcription of *Period* (*Per*) and *Cryptochrome* (*Cry*) is driven by a complex of basic helix-loop-helix transcriptional factors, including circadian locomotor output cycles kaput (CLOCK) and Aryl hydrocarbon receptor nuclear translocator-like 1 (BMAL1), and repressed by PER protein-mediated negative feedback. The SCN comprises a heterogeneous population of cells that differ in endogenous period, pacemaking ability, and expression of clock genes and neuropeptides (Honma et al., 2012; Hastings et al., 2014). In the SCN, a large population of different cells is synchronized to produce a coherent, robust rhythm.

Circadian timing systems, like most physiological processes, cannot escape the effects of aging. Aging is commonly associated with changes in the quantity and quality of sleep, and with specific sleep disorders, including sleep episode fragmentation, in humans and other mammals (Bliwise, 1993; Turek et al., 1995; Van Someren, 2000). In rodents, aging induces changes in locomotor activity rhythms, including decreased amplitude, increased fragmentation, shortened or lengthened free-running period, slower re-entrainment following LD cycle shifts, and altered light sensitivity (Pittendrigh and Daan, 1974; Turek et al., 1995; Zhang et al., 1996; Scarbrough et al., 1997; Valentinuzzi et al., 1997). The age-related decline in behavioral rhythmicity can be partially reversed by implanting fetal SCN tissue into the third ventricle of aged animals (Van Reeth et al., 1994; Viswanathan and Davis, 1995; Cai et al., 1997; Li and Satinoff, 1998), indicating that the SCN plays an important role in age-related behavioral changes.

Several studies have examined the effects of aging on rhythmic expression of clock genes in the SCN, with conflicting results (Asai et al., 2001; Yamazaki et al., 2002; Kolker et al., 2003, 2004; Wyse and Coogan, 2010; Nakamura et al., 2011). Some studies have found evidence of age-related disruption of *Bmal1* and *Clock* (Kolker et al., 2003; Wyse and Coogan, 2010), but most have reported that clock genes, such as *Per* genes, remain unaltered in aged animals (Asai et al., 2001; Yamazaki et al., 2002; Kolker et al., 2004; Davidson et al., 2008; Nakamura et al., 2011; Sellix et al., 2012). For example, Asai et al. (2001) measured expressions of *Per1*, *Per2*, and *Cry1* in the SCN and found that the waveforms of transcript rhythmicity were identical in aged (22- to 26-month-old) rats and young (2- to 3-month-old) rats. Similarly, an *ex vivo* study (Yamazaki et al., 2002), which measured free-running rhythms in the *Per1-luciferase (Per1-luc)* rat model, confirmed that the waveforms of *Per1* rhythmicity were identical in young (2-month-old) animals and aged (24- to 26-month-old) animals, although the free-running circadian period was shorter in aged animals. Therefore, with the exception of *Bmal1* and *Clock*, there is little evidence that aging impacts the expression of clock genes, although a substantial age-related impact on behavioral rhythmicity has been observed.

In an effort to understand the minimal impact of aging on the rhythms of key mammalian clock genes, we considered whether entrainment to LD cycles might mask some age-induced deficiencies of molecular clocks. We hypothesized that any lack of robustness in SCN oscillators in aged animals would more likely be revealed under free-running conditions in the absence of LD cycles, which would likely boost the amplitude of molecular rhythms and help maintain interoscillator synchrony. The effects of aging on clock genes in the SCN have previously been examined (Asai et al., 2001; Yamazaki et al., 2002; Kolker et al., 2003, 2004; Wyse and Coogan, 2010; Nakamura et al., 2011), but animals were housed under LD cycles or only briefly held in constant dark (DD) conditions. In the present study, we performed *ex vivo* bioluminescent imaging of cultured SCN slices of young and aged PER2::luciferase knock-in (PER2::LUC) mice housed in prolonged DD conditions (10 d). We observed the effects of aging on PER2::LUC oscillations in the SCN population, and we recorded PER2 oscillations at the single-cell level using an electron-multiplying (EM) charge-coupled device (CCD) camera. Mathematical analysis of the PER2 cellular oscillations revealed a longer circadian periodicity and desynchronized cellular rhythms in the aged SCN.

## Materials and Methods

### Animals

Male heterozygous PER2::LUC mice (C57BL/6J background; http://jaxmice.jax.org/strain/006852.html) were obtained from The Jackson Laboratory and bred for use in these experiments. Animals were classified into young and aged groups, and were housed separately until they reached the age for experimentation. Animals were maintained under controlled environmental conditions (temperature, 22 ± 2°C; LD cycle, 12 h light: 12 h dark, 100–300 lux at the bottom of the cage) with food and water available *ad libitum*. All animal procedures were performed according to the regulations of University of California Los Angeles Institutional Animal Care and Use Committee.

### Real-time monitoring of bioluminescence by photomultiplier tube

Young (3- to 5- month-old *n* = 10) and aged (13- to 15- month-old *n* = 10) PER2::LUC mice were used in this experiment. Upon reaching the age of experimentation, animals were individually housed; each cage contained a running wheel. Locomotor activity was measured as running wheel revolutions recorded in 1 min bins and analyzed with ClockLab Software (Actimetrics). Half of the young and aged mice were kept under a normal 12 h light: 12 h dark LD cycle (light-on time, 6:00 A.M.) for at least 2 weeks, and then the animals were killed at zeitgeber time (ZT) 10-11 (ZT12 is defined as the time of lights off). The remaining young and aged animals were kept in DD for 10 d with monitoring of their wheel-running activities. The time of activity onset was calculated for individual animals on the day before the sampling and then they were killed in a dark room with dim red light (<1 lux) at circadian time (CT) 10-11 (CT12 is defined as the time of activity onset). The time of day, at which we harvested the tissue and cultured it, has little effect on its subsequent phase.

Bioluminescence monitoring was performed according to previously reported procedures (Yamazaki et al., 2002; Yoo et al., 2004; Savelyev et al., 2011). The brain of each mouse was removed, placed in chilled HBSS, and sliced in the coronal plane on a Microslicer (Dosaka EM) at a thickness of 300 µm. The bilateral SCN and a minimum of surrounding tissue were isolated from the slice using scalpels. The explants were placed in a LumiCycle equipped with photomultiplier tubes (PMTs; Actimetrics) and kept inside a light-tight 36.5°C environmental chamber. Recordings were started at ZT12 or CT12 for LD-housed mice and DD-housed mice, respectively. The bioluminescence signal was counted in 1 min bins every 10 min for at least 6 d without changing the media. The data were detrended by subtraction of the 24 h running average from the raw data and then smoothed with a 2 h running average (Origin Lab Software). The peak phase was set at the highest point of the smoothed data in each cycle. The amplitude was determined using the highest and lowest points of each cycle.

### EM-CCD camera recording of bioluminescence in individual SCN cells

Young (3- to 5- month-old; *n* = 6) and aged (20- to 24- month-old; *n* = 6) mice were used in this experiment. Animals were transferred to DD conditions for 10 d. SCN explants were prepared using the procedure described for PMT recording. The SCN slices were kept in a light-tight 36.5°C environmental chamber and imaged using an ImagEM EM-CCD camera (Hamamatsu Photonics; frame rate, 32 frames/s; EM gain, 1200; objective lens; UPlanSApo 10×; numerical aperture, 0.40; Olympus). The bioluminescence images were stored as consecutive 50 s summed images every 1 min for 5 d (7200 images), with recording starting at CT12. One hundred twenty-eight images were averaged to produce a series of 60 min sampling images (AQUACOSMOS Software; Hamamatsu Photonics). Fifty cells in the SCN were randomly selected from a single image as regions of interest (ROIs). In the ROIs, brightness values were measured and tracked for all 120 images.

### Data analysis for single SCN cell monitoring

To quantify the phase and amplitude of the cellular rhythms, bioluminescence signals extracted from the ROIs of 120 images were analyzed using standard techniques. From simultaneous measurements of 50 cellular signals {*x_i_*(*t*): *t* = 1, 2…, 120; *i* = 1, 2…, 50}, the acrophases were obtained as follows. First, at time *t*, phase θ*_i_*(*t*) of the *i*th cell was determined by Cosinor’s method (Halberg et al., 1967). Namely, the bioluminescence signal {*x_i_*(*s*): *s* = *t* − 14,…, *t* + 14} of the *i*th cell in the time interval from *s* = *t* − 14 h to *s* = *t* + 14 h was fitted to a cosine function $a_{i}(t)*\cos\left(\frac{2\pi \;(s+\theta_{i}(t))}{24}\right)$, where the oscillation period was fixed to 24 h; *a_i_*(*t*) represents the oscillation amplitude. The estimated phase θ*_i_*(*t*) was considered reliable when the coefficient of determination was >0.8. The initial transient and final decaying portions were removed from the 120 h recordings, and the phase θ*_i_*(*t*) was obtained for the time interval from *t* = 27 h to *t* = 98 h. Next, with respect to the mean bioluminescence signal $\left\{\frac{1}{50}{\sum_{i=1}^{50}x}_{i}\left(s\right):s=t-14,\cdots ,t+14\right\}$, which was obtained by averaging the measurement across all 50 cells, the corresponding mean phase $\overset{®}{\theta }(t)$ was determined in the same manner as for individual cells. By subtracting the mean phase $\overset{®}{\theta }(t)$, the acrophase was obtained for each cell, as $\varphi_{i}(t)=\theta_{i}(t)-\overset{®}{\theta }(t)$. To determine the level of synchrony among the 50 cells in each slice, the SD of the 50 acrophases was calculated. An alternative index for measuring synchrony (Bernard et al., 2007) yielded essentially identical results, indicating that the obtained result was not sensitive to the choice of the synchronization quantity. To measure the cellular amplitude, the coefficients {*a_i_*(*t*): *i* = 1, 2,…, 50} determined in the Cosinor’s analysis of the cellular signals were used. The time sequence of the amplitudes was then normalized as ${\overset{∼}{a}}_{i}\left(t\right)=a_{i}(t)/a_{i}(1)$, which sets the initial amplitude at unity. For each slice, the SD of the 50 cellular amplitudes was then calculated. To examine period lengthening, peak-to-peak periods were determined; peaks were located using the Cosinor’s fitting of the cellular signals. The period ratio between the first and last cycles was then calculated.

### Statistical analysis

In PMT recording and EM-CCD camera recording, datasets of phase and amplitude were analyzed by two-way repeated-measures ANOVA to examine the interaction effect of age and cycle (time) on the PER2::LUC rhythms. When the ANOVA detected significance, the *post hoc* Bonferroni method was used to examine the difference between young and aged groups at each time point. In EM-CCD camera recording, the difference in change in the free-running circadian period between young and aged groups was examined by unpaired Student’s *t* test. Statistical analysis was performed by IBM SPSS Statistics software. All results are presented as the mean ± SD and were considered significant at *p* < 0.05.

## Results

**Figure 1.**
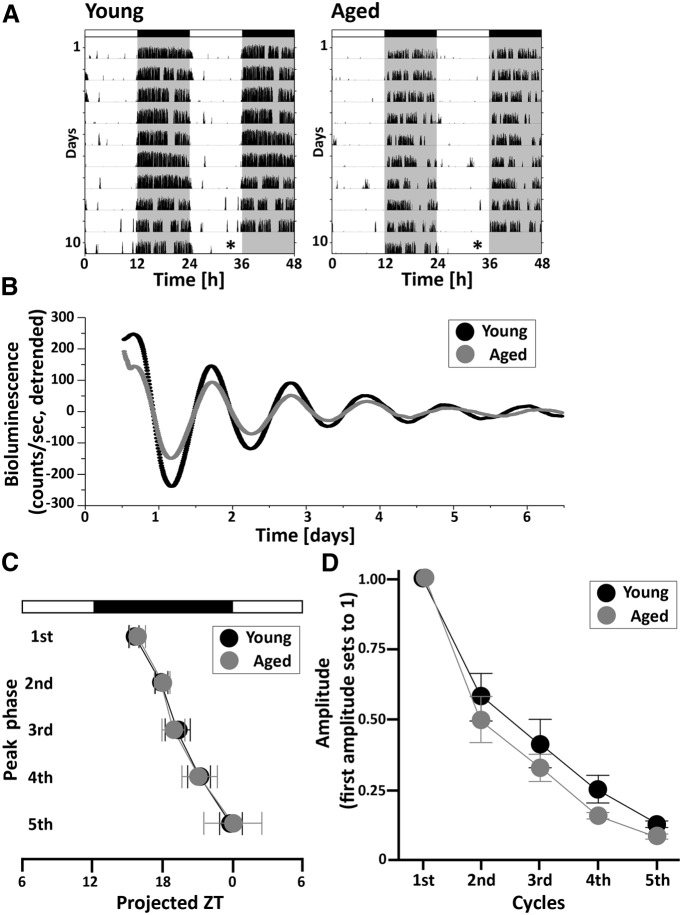
Effects of aging on PER2::LUC rhythms in SCN explants from mice maintained under LD cycles. ***A***, Representative double-plotted actograms of wheel-running activity in young and aged PER2::LUC mice maintained under LD cycles. Lighting conditions are indicated at the top of the figure; open bars indicate light phases, and closed bars indicate dark phases. Asterisks indicate the timing of the killing of the animals. ***B***, Typical examples of the PER2::LUC rhythm of SCN explants from mice maintained under LD cycles, as measured by PMT. ***C***, Phase map of peak PER2::LUC rhythms in the SCN. Each point represents the average peak of PER2::LUC rhythms in each cycle, plotted relative to the LD cycle prior to the killing of the animals. ***D***, Amplitude of PER2::LUC rhythms in the SCN. The amplitude of each oscillation was determined as the sum of the absolute values of the lowest and highest points (counts). Data are shown as the mean ± SD. *n* = 5 per group.

First, we recorded bioluminescence rhythms in SCN explants from mice maintained under normal LD cycles (Fig. 1). The wheel-running activity of young and aged mice housed under LD cycles was monitored for at least 10 d, and then the mice were killed at ZT10-11 (Fig. 1*A*). Both young and aged SCN tissue clearly showed circadian rhythms in PER2::LUC expression (Fig. 1*B*). We calculated the phase and amplitude of each cycle over time. Two-way repeated-measures ANOVA revealed no interaction between age and cycle at either phase (*p* = 0.966^a^) or amplitude (*p* = 0.112^b^) data (Fig. 1*C*,*D*, Table 1).

**Table 1.** Statistical table: results of two-way repeated-measures ANOVA for datasets of phase and amplitude in PMT recordings (Figs. 1C,D, 2C,D), and datasets of phase spread and amplitude in EM-CCD camera recordings (Fig. 3C,D)

		Interaction effect [age × cycle (time)]		
		*F* value	*P*-value	Figure
a	Phase in LD	*F*_(4,32)_ = 0.141	0.966	1C
b	Amplitude in LD	*F*_(4,32)_ = 2.045	0.112	1D
c	Phase in DD	*F*_(4,32)_ = 11.328	<0.001	2C
d	Amplitude in DD	*F*_(4,32)_ = 12.377	<0.001	2D
e	Phase spread in camera	*F*_(71,710)_ = 3.968	<0.001	3C
f	Amplitude in camera	*F*_(71,710)_ = 0.997	0.488	3D

**Figure 2.**
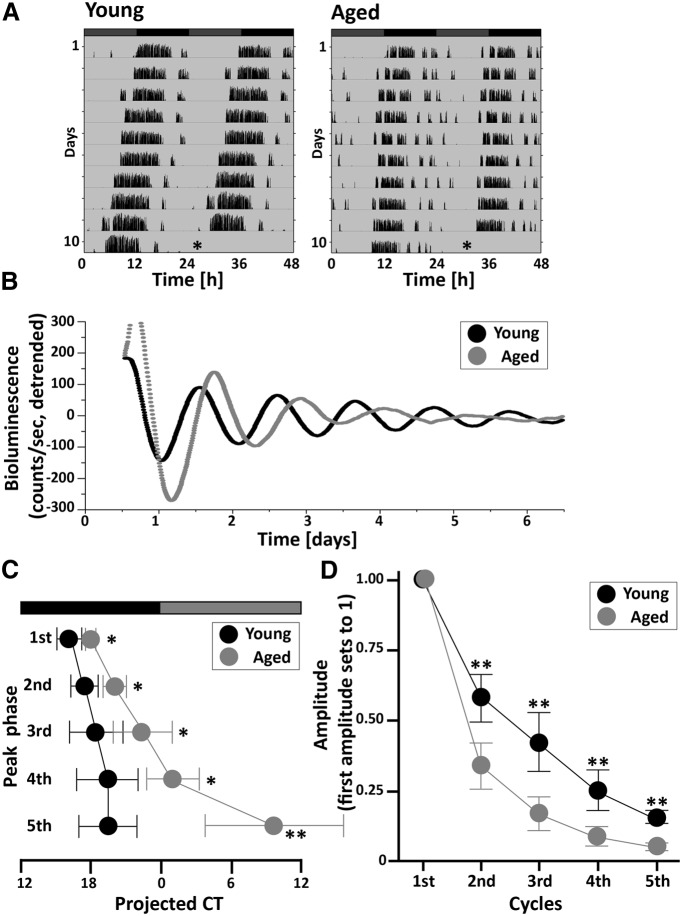
Effects of aging on PER2::LUC rhythms in SCN explants from mice maintained in DD. ***A***, Representative double-plotted actograms of wheel-running activity in young and aged PER2::LUC mice maintained in DD. Asterisks indicate the timing of the killing of the animals. ***B***, Typical examples of PER2::LUC rhythms of SCN explants from mice maintained in DD for 10 d, as measured by PMT. ***C***, Phase map of peak PER2::LUC rhythms in the SCN. Each point represents the average peak of PER2::LUC rhythms in each cycle, plotted relative to the circadian time prior to the killing of the animals. ***D***, Amplitude of PER2::LUC rhythms in the SCN. The amplitude of each oscillation was determined as the sum of the absolute value of the lowest and highest points (counts). Data are shown as the mean ± SD. *n* = 5 per group. *p < 0.05, **p < 0.01 for young vs. aged animals (Bonferroni *post hoc* comparison).

Next, we recorded bioluminescence rhythms in SCN explants from mice maintained in DD (Fig. 2). The wheel-running activity of young and aged mice in DD was monitored for 10 d, and the mice were then killed in the dark at CT10-11 (Fig. 2*A*). Both young and aged SCNs clearly showed circadian rhythms of PER2::LUC expression (Fig. 2*B*). Two-way repeated-measures ANOVA showed an interaction between age and cycle in the peak phases (Fig. 2*C*, Table 1; *p* < 0.001^c^). The phase differences gradually increased up to the fifth cycle [peak phase (CT); 16.13 ± 1.24 and 18.03 ± 0.63 for the first cycle (*p* < 0.05^g^); 17.53 ± 1.77 and 20.13 ± 1.40 for the second cycle (*p* < 0.05^h^); 18.43 ± 2.34 and 22.33 ± 2.68 for the third cycle (*p* < 0.05^i^); 19.50 ± 2.89 and 24.80 ± 2.31 for the fourth cycle (*p* < 0.05^j^); 19.7 ± 2.67 and 33.23 ± 6.36 for the fifth cycle (*p* < 0.01^k^) in young and aged SCNs (*n* = 5 per group), respectively; Fig. 2*C*, Table 2]. As indicated by the delayed peaks, the free-running period of PER2::LUC rhythm in aged SCNs was significantly longer than in young SCNs. Moreover, the amplitude was decreased by the aging; two-way repeated-measures ANOVA revealed a significant interaction between age and cycle in the amplitude data (Fig. 2*D*, Table 1; *p* < 0.001^d^). Significant differences between young and aged groups were detected in the second to the fifth cycle [normalized amplitude (first cycle set to 1.00); 0.60 ± 0.12 and 0.35 ± 0.07 for the second cycle (*p* < 0.01^l^); 0.39 ± 0.10 and 0.18 ± 0.06 for the third cycle (*p* < 0.01^m^); 0.25 ± 0.07 and 0.10 ± 0.03 for the fourth cycle (*p* < 0.01^n^); 0.15 ± 0.06 and 0.05 ± 0.02 for the fifth cycle (*p* < 0.01^°^) in young and aged SCNs (*n* = 5 per group), respectively; Fig. 2*D*, Table 2].

**Table 2.** Statistical table for pairwise comparisons

	Data structure	Type of test	*p* value	Figure
g	Normally distributed	Bonferroni	0.016	2C
h	Normally distributed	Bonferroni	0.033	2C
i	Normally distributed	Bonferroni	0.040	2C
j	Normally distributed	Bonferroni	0.012	2C
k	Normally distributed	Bonferroni	0.002	2C
l	Normally distributed	Bonferroni	0.003	2D
m	Normally distributed	Bonferroni	0.004	2D
n	Normally distributed	Bonferroni	0.003	2D
o	Normally distributed	Bonferroni	0.004	2D
p	Normally distributed	*t* test	0.026	3E

**Figure 3.**
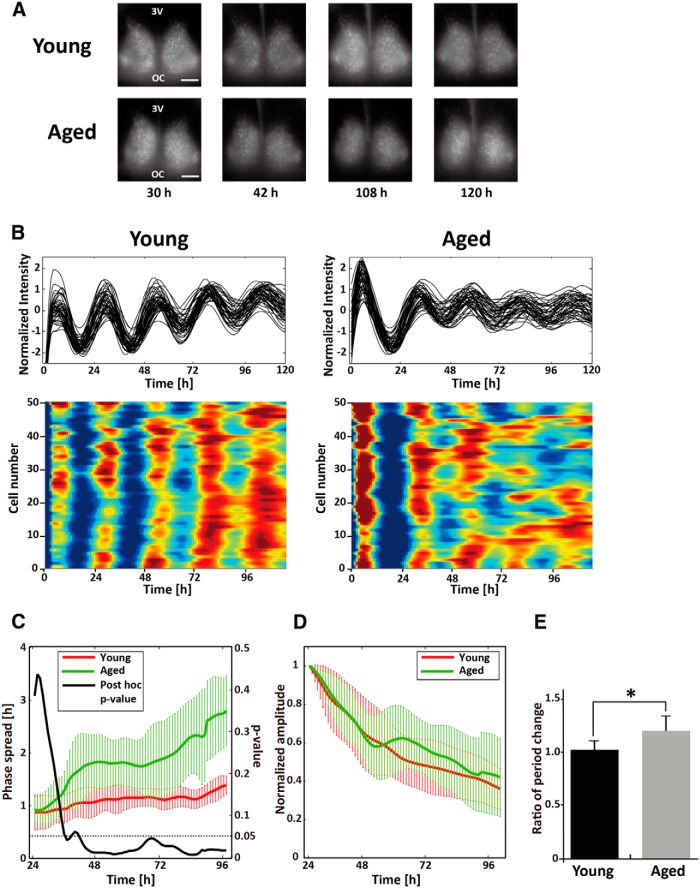
Effects of aging on PER2::LUC rhythms in SCN explants from mice maintained in DD. ***A***, Representative images of PER2::LUC in SCN explants collected from young and aged mice maintained in DD for 10 d, recorded by an EM-CCD camera at 30, 42, 108, and 120 h after the start of recording. ***B***, Representative graphical (top) and raster (below) plots of PER2::LUC rhythms of individual cells in SCN explants of young and aged SCNs. ***C***, ***D***, Phase spreads (***C***) and normalized amplitudes (***D***) of PER2::LUC rhythms averaged over young (red) and aged (green) SCNs are shown over five cycles. ***E***, Ratios of free-running circadian period change from the first to the fifth cycle of PER2::LUC rhythms in 50 individual SCN cells are shown. Data are shown as the mean ± SD. *n* = 6 per group. *p < 0.05 (*t* test). Scale bar, 100 μm.

Finally, we recorded PER2::LUC rhythms in individual SCN cells of aged mice using an EM-CCD camera. Young and aged mice were housed in DD for 10 d and then killed in darkness at CT10-11. The high resolution of the camera system allowed us to capture images of individual PER2::LUC rhythms in SCN explants and to track individual cells over five cycles (120 h; Fig. 3*A*,*B*). Although PER2::LUC rhythms in individual cells showed clear circadian rhythms in both young and aged SCNs, aged cells gradually drifted out of phase with each other. To analyze this asynchrony, we calculated the SD of the acrophases of the 50 cells, as an index of their phase spread (Fig. 3*C*). The phase spread in young SCNs was <1 h during the first cycle (<24 h) and remained <1.5 h until the fifth cycle (>96 h). In contrast, the phase spread in aged SCNs showed a 1 h difference during the first cycle (<24 h) and gradually increased, until it exceeded 2 h by the fifth cycle (>96 h). Two-way repeated-measures ANOVA indicated a significant interaction effect between age and time in the phase spread (Table 1; *p* < 0.001^e^). Bonferroni *post hoc* comparison detected the difference between young and aged SCNs, and revealed that the difference became significant in recording time from 39 to 40 h, and it remained significant after 44 h (Fig. 3*C*). We next calculated the amplitude of PER2::LUC rhythm in individual cells of SCN explants from young and aged mice over five cycles. In both age groups, the amplitude gradually decreased; two-way repeated-measures ANOVA revealed no significant interaction effect between age and time in the amplitudes (Fig. 3*D*, Table 1*; p* = 0.488^f^). Finally, we calculated the changes in the free-running circadian period of individual SCN cells. The change in circadian period differed significantly between SCNs from young and aged mice [ratio of period change: 1.04 ± 0.09 and 1.20 ± 0.14 in SCNs from young and aged mice (*n* = 6 per group), respectively (*p* < 0.05^p^); Fig. 3*E*, Table 2].

## Discussion

Aging profoundly alters circadian behavior and physiology. However, the molecular clockwork that underlies these circadian rhythms appears to be more resilient. For example, most studies of oscillations in *Period* genes have failed to detect substantial age-related decline. In our first experiment, we examined PER2::LUC rhythms in the SCN of mice housed under normal LD cycles. Under these conditions, we observed only a small difference between young and aged SCN explants in PER2::LUC amplitude, and no difference was observed in the peak phase (Fig. 1). These observations are consistent with a previous study that examined PER2::LUC mice using immunohistochemistry (Nakamura et al., 2011). Past work (Yamazaki et al., 2002) using a *Per1-luc* rat did find that aged SCN explants exhibited a shorter free-running period than those from young animals, without changes in waveform or amplitude. Other studies used *in situ* hybridization and immunohistochemistry techniques to characterize subtle age-related changes in the rhythmic expression of *Per* genes (Asai et al., 2001; Kolker et al., 2003, 2004; Nakamura et al., 2011). Notably, *Clock* and *Bmal1* expression was reduced in SCN tissue of aged hamsters that were housed under LD cycles and killed after 3 d in DD (Kolker et al., 2003). A more recent study reported a reduction in BMAL1 and CLOCK proteins in the SCN of old mice (Chang and Guarente, 2013). These data may indicate that the positive activators (*Clock* and *Bmal1*) of the molecular clock are more strongly influenced by aging than the negative-feedback elements (*Per* and *Cry*).

It is well established that *Per1* and *Per2* are induced in the SCN after light exposure during the night (Shearman et al., 1997; Shigeyoshi et al., 1997); thus, it is possible that daily light exposure masks the true impact of aging on PER2::LUC rhythms in the SCN. In support of this hypothesis, we found that both the amplitude and peak phase of PER2::LUC rhythms in the SCN of mice housed in DD conditions for 10 d (DD-housed mice) were influenced by aging. The PMT allows us to measure the summed response of the SCN circuit. We observed a lengthened free-running period and an attenuated waveform in PER2::LUC rhythms in SCN explants of aged DD-housed mice (Fig. 2). We emphasize that the critical difference between the present study and the previous studies (Asai et al., 2001; Yamazaki et al., 2002; Kolker et al., 2003, 2004; Nakamura et al., 2011) is the use of an LD environment. Previous studies did not detect age-related changes in rhythmic expression of *Per1* and *Per2* genes (Asai et al., 2001; Yamazaki et al., 2002; Kolker et al., 2003, 2004; Nakamura et al., 2011) in animals housed in LD cycles or immediately after release to the DD condition (e.g., 2 or 3 d in DD). In contrast, our DD-housed mice were not exposed to light for at least 10 d prior to preparation of the slice cultures.

In our experiment on young mice, the free-running period of PER2::LUC rhythms in SCN explants of the LD-housed mice was longer than that of the DD-housed mice. This indicates that the LD condition lengthened the free-running period of the PER2::LUC rhythms. To measure the free-running period in the behavioral rhythm of mice, environmental factors, which may influence the rhythm, should be eliminated. For this reason, mice are usually maintained in DD conditions for the observation of their intrinsic rhythms. In the present study, we took the same approach to observe the effects of aging. Previous reports showed that aging lengthens the free-running period in C57BL/6J mice, which have the same background line of PER2::LUC mice (Turek et al., 1995). Moreover, the period length of the behavioral rhythm in these mice is known to be consistent with that of the SCN molecular rhythm (Liu et al., 2007). Taken together, these findings suggest that the elimination of light for 10 d reveals the intrinsic effect of aging on the free-running period of PER2::LUC rhythm in the SCN.

Next, we used camera recordings to examine the rhythms of individual neurons. In the camera recordings, we found that aging lengthened the free-running period of the PER2::LUC rhythms of individual SCN cells within the explant, but did not affect the amplitude (Fig. 3). Moreover, the single-cell analysis revealed that the phase of PER2::LUC rhythms in individual SCN cells became dissociated with age (Fig. 3). Considering the period length and the waveform separately, these data suggest that aging lengthens the period of PER2::LUC rhythms in individual SCN cells, which results in lengthening of the period in the entire SCN. In contrast, the attenuation of the waveform appears to be a population phenomenon due to the phase dissociation of the PER2::LUC rhythms among individual SCN cells. We used older aged animals (20- to 24- month-old) in the single-cell recordings than those we used (13- to 16- month-old) in PMT recordings to maximize our chances of seeing an impact of aging on single SCN cells. Several prior studies that examined the effect of aging on the rhythms of *Per* genes in the SCN (Asai et al., 2001; Yamazaki et al., 2002; Kolker et al., 2003, 2004) used relatively old animals (17- to 26- month-old) and did not detect age-related changes in rhythmic expression of *Per1* and *Per2* genes. In contrast, in the present study, the PMT recordings revealed an age-related decline in PER2::LUC rhythms at the SCN population level even with middle-aged mice (13- to 16- month old). A recent behavioral analysis found that the free-running period of 10-month-old mice was significantly longer than that of 3-month-old mice (Farajnia et al., 2012). Decline in the total amount of activity was also observed with the older mice. The changes in the free-running period and the total amount of activity declined further with older mice (Farajnia et al., 2012). In addition, previous *in vivo* multiunit neuronal activity recordings in freely moving middle-aged (13-month-old) mice indicated that circadian rhythms of electrical activity in the SCN are already degraded by middle age (Nakamura et al., 2011). These previous studies as well as the present results suggest that the phase dissociation of the PER2::LUC rhythms among individual SCN cells is observed not only in aged (20- to 24-month-old) mice, but also in middle-aged (13- to 16-month-old) mice, which we used for the PMT recordings.

In our PMT recordings of DD-housed mice, the initial peak of PER2::LUC rhythm in SCNs from aged mice was found to be higher than that in SCNs from young mice (Fig. 2). The camera recordings also indicated that the initial peak of PER2::LUC was well synchronized in individual cells of aged SCNs, relative to those of SCNs from young mice (Fig. 3). Culture preparation might have affected the phase of individual cells, although the SCNs of young PER2::LUC mice are not usually affected by culture preparation (Davidson et al., 2009). If intercellular coupling is weaker in SCNs from aged mice, the culture preparation might have a stronger effect on the synchronization of cellular clocks in individual SCN cells.

Although the present study suggests weaker intercellular couplings in aged SCNs, the mechanisms that underlie these age-related changes in the SCN are still unclear. The cellular oscillators within the SCN are coupled via neuropeptides (Welsh et al., 2010; Maywood et al., 2011), and there is evidence of age-related decline in the expression of key neuropeptides (Kawakami et al., 1997; Krajnak et al., 1998; Duncan et al., 2001; Aton and Herzog, 2005; Maywood et al., 2011). Furthermore, GABA is the major transmitter within the SCN network and prior work has shown that the number of GABAergic synaptic terminals in the SCN is diminished in old mice (Palomba et al., 2008). Physiological recordings have confirmed that GABAergic postsynaptic currents are reduced in amplitude and frequency in the aging SCN (Nygård et al., 2005; Farajnia et al., 2012). *In vivo* and *in vitro* experiments from several laboratories have shown that the electrical activity rhythms that characterize SCN physiology are also vulnerable to the effects of aging (Satinoff et al., 1993; Watanabe et al., 1995; Aujard et al., 2001; Nakamura et al., 2011; Farajnia et al., 2012). A reduction in electrical activity would be expected to reduce the release of peptides as well as GABA. Finally, in a recent study, patch-clamp recordings of SCN neurons in aged mice revealed that the circadian modulation of large-conductance Ca^2+^-activated K^+^ (BK) channel activity was lost because of a reduction in BK currents during the night (Farajnia et al., 2015). Thus, we favor the hypothesis that aging leads to changes in electrical activity, which, in turn, weaken intercellular coupling among SCN cells.

In summary, our data indicate that, at the population level, aging lengthens the free-running period and attenuates the amplitude of population-based PER2::LUC rhythms in the SCN. At the single-cell level, we found that aging lengthens the period and reduces the synchrony of single-cell oscillations without reducing their amplitude. Under a robust LD cycle, these deficits are masked; thus, our research is consistent with the suggestion that exposure to a robust LD cycle will help to maintain the strength of circadian oscillations in elderly people. Indeed, there is already some evidence that reinforcing the circadian system using light can improve daytime alertness and sleep quality in aged individuals (Mishima et al., 2001; Riemersma-van der Lek et al., 2008; Lieverse et al., 2011; Friedman et al., 2012; Royer et al., 2012). However, other studies have observed only small changes in sleep as a result of light therapy (Ancoli-Israel et al., 2002; Dowling et al., 2008; Friedman et al., 2009). The design of many of these studies should be re-evaluated in the light of our current understanding of the light-input pathways of the circadian system and, in particular, the importance of the photopigment melanopsin (LeGates et al., 2014). Although aging in mice may well differ from aging in humans, our analysis reveals a hidden vulnerability in the molecular clockwork that indicates that the aging circadian system is more sensitive to environmental challenges than previously suspected. We speculate that this vulnerability may play a role in the sleep and circadian dysfunction that is so common in the aging population. If correct, these findings reinforce the importance of living in a temporally structured environment.

## Synthesis

The decision was a result of the Reviewing Editor Rae Silver and the peer reviewers coming together and discussing their recommendations until a consensus was reached. A fact-based synthesis statement explaining their decision and outlining what is needed to prepare a revision is listed below. The following reviewers agreed to reveal their identity: Nicolas Cermakian, Ruud Buijs

General comments:

The paper clearly demonstrates deterioration of the SCN network in aged mice when the animals are housed in DD conditions. On the one hand, this in contrast when recording of SCN PER luciferase is done in SCN slices from animals housed in LD. On the other hand, the results are consistent with long established data showing that increased light exposure in an LD situation amplifies the day night rhythm in activity.

Major comments:

• The authors compare clock gene expression in the SCN of young and aged mice. They hypothesize that the small changes of SCN clock gene expression (and the sometimes conflicting results) seen in the literature are due to the LD or short-term DD conditions of those studies, and that a LD cycle could mask more extensive effects of aging on SCN clock gene expression. For this reason, they used SCN explants from PER2::LUC mice held either in LD or for 10 days in DD, and recorded whole tissue or single-cell bioluminescence. While the young/aged differences are minor in LD vs DD mice, there are clear age-dependent differences in phase and amplitude at the whole tissue level, and increased phase spread (but little amplitude or period changes) at the single-cell level in aged mice. The extent and mechanisms of circadian disruption upon aging is a matter of substantial interest. This is a well-performed study, and the data are clear, and this is the major contribution of the work

• Although the LD-DD condition differences are very clear, we are not entirely convinced that LD vs. DD is what distinguishes this study from previous studies, as some had also used DD conditions in previous work cited in the paper (e.g. Kolker et al, 2003, 2004, Nakamura et al, 2011); differences in experimental conditions, mouse strains, etc., could also be involved.

• Although this study is well performed and the results are clear and interesting, it is not entirely surprising that a LD cycle would strengthen rhythms in the SCN, reduce neuron phase dispersion, and increase amplitude.

• Also, this study does not bring any new data on the possible mechanisms underlying the age-related changes in SCN rhythmicity - a point that should be made inhte discussion.

• All statistics are Student t test. This is not appropriate for time series. In data sets comparing young and aged mice over several days (Figs 1C-D, 2C-D, 3C-D), two way ANOVAs must be used, and if a significant difference is found in the ANOVA, pairwise comparisons can then be done using posthoc tests. All stats must be redone, and the text and figures revised accordingly.

Minor comments:

• Page 6: For the non-chronobiologist readers, ZT and CT should be explained. Moreover, the authors should explain that determination of CT10-11 was based (hopefully) on the locomotor activity rhythms of each mouse's activity record independently, and that all mice were therefore sacrificed 1-2h prior to their particular time of activity onset. It would also be good to mention or reassure the reader that at this time of day, harvesting the tissue and culturing it has little effect on its clock phase.

• Pages 6-7: Were the slices used for the camera pre-tested in the lumicycle? It is unclear why the aged mice used for the Lumicycle and the single-cell experiments have such different ages (13-16 months vs. 20-24 months). Given this, can one really relate the results of the two experiments, i.e. using the single-cell data to explain the whole-tissue data, as the authors do? This should be discussed. It would also be good to add to this a discussion of the age of the mice they used vs. the age of the animals used in previous studies.

• Page 11, 3 lines from bottom: "Finally, we calculated the changes in the free-running circadian period of individual SCN cells, with respect to period change." Consider revising (repetition).
